# Advance in Research on *Mycobacterium tuberculosis* FabG4 and Its Inhibitor

**DOI:** 10.3389/fmicb.2018.01184

**Published:** 2018-06-06

**Authors:** Debajyoti Dutta

**Affiliations:** Department of Biochemistry, Faculty of Medicine and Dentistry, University of Alberta, Edmonton, AB, Canada

**Keywords:** FabG4, *Mycobacterium tuberculosis*, biofilm, β-oxo acyl-ACP reductase, inhibitor

## Abstract

Increasing evidence from recent reports of drug-resistant mycobacterial strains poses a challenge worldwide. Drug-resistant strains often undergo mutations, adopt alternative pathways, and express drug efflux pumps to reduce or eliminate drug doses. Besides these intrinsic resistance mechanisms, bacteria can evade drug doses by forming biofilms. Biofilms are the concerted growth of adherent microorganisms, which can also be formed at the air-water interface. The growth is supported by the extracellular polymer matrix which is self-produced by the microorganisms. Reduced metabolic activity in a nutrient-deficient environment in the biofilm may cause the microorganisms to take alternative pathways that can make the microorganisms recalcitrant to the drug doses. Recent works have shown that *Mycobacterium tuberculosis* expresses several proteins during its growth in biofilm, those when deleted, did not show any effect on mycobacterial growth in normal nutrient-sufficient conditions. Studying these unconventional proteins in mycobacterial biofilms is therefore of utmost importance. In this article, I will discuss one such mycobacterial biofilm-related protein FabG4 that is recently shown to be important for mycobacterial survival in the presence of antibiotic stressors and limited nutrient condition. In an attempt to find more effective FabG4 inhibitors and its importance in biofilm forming *M*. *tuberculosis*, present knowledge about FabG4 and its known inhibitors are discussed. Based on the existing data, a putative role of FabG4 is also suggested.

## Introduction

The ability of *Mycobacterium tuberculosis* to form biofilms was noted almost 120 years ago (Jones, 1896). However, the physiological and molecular basis of biofilm is only beginning to unravel until recently. The first protein that was found to be involved in *M*. *smegmatis* maturation of biofilm is the chaperone GroEL-1 (Ojha et al., 2005). GroEL-1 deficient mutant of *M*. *smegmatis* was suggested to lack GroEL-1 interaction with fatty acid synthesis type -II complex thereby reducing the mycolic acid content in biofilms. Mycolic acids are the predominant components of mycobacterial cell envelope that are produced by fatty acid synthesis type-II pathway in mycobacteria. The reason that mycobacterial cell envelope largely contributes to the biofilm attachment endorses mycolic acids may be involved in biofilm formation (Marrakchi et al., 2014). However the physiology of mycobacteria changes as it shifts from planktonic growth to biofilm dependent growth and results in several modifications in the expression level of protein and molecules pertaining to the cell envelope (Ojha et al., 2008; Sambandan et al., 2013; Rastogi et al., 2017). Fatty acid synthesis and its associated pathways for mycobacterial cell envelope synthesis are one of the major areas for developing antitubercular drugs (Zumla et al., 2013). Because of this altered phenotypes and a waxy extra-cellular matrix of biofilm, Mycobacteria become resilient to drug doses (Islam et al., 2012). Therefore the conventional TB drugs may not be as effective for biofilm-forming mycobacteria.

Membrane and cell envelope-associated biofilm-related proteins are particularly of interest as these proteins are likely to be involved in the making of cellular attachment to the biofilms. For example, recent studies have shown that a lipid transporter MmpL11 is specifically required for biofilm formation (Pacheco et al., 2013). Other works have identified a number of proteins specific to mycobacterial growth in the biofilm at air-water interface (Kerns et al., 2014). One of the proteins that are conserved among mycobacterial species is FabG4. The protein was proposed to possess host antigenic property and has a potential to be a biofilm-specific marker (Kerns et al., 2014). In addition to that, FabG4 was recognized as one of the crucial protein for mycobacterial survival in a stressed condition. This article will discuss the known facts about FabG4, its inhibitors, and discuss its possibility to serve as a candidate to study and treat biofilm-related mycobacteria.

## Importance of FabG4 in Mycobacteria

FabG commits the second step of fatty acid synthesis that is to convert β-oxo acyl-ACP to β-hydroxy acyl-ACP. *M*. *tuberculosis* genome contains multiple FabG homologs. Two of them are conserved among all mycobacterial species, FabG1 and FabG4. FabG1 remain at the focus of attention because it takes part in fatty acid synthesis type-II (Marrakchi et al., 2002). On the other hand, several studies have indicated that FabG4 is not an inactive gene in the *M*. *tuberculosis* genome. Its expression was first documented in the proteome by using 2-D gel electrophoresis accompanying MALDI-MS analysis (Jungblut et al., 1999) and later verified by others (Rosenkrands et al., 2000; Sinha et al., 2002). Gu et al. (2003), first provide the evidence that the protein is expressed in the mycobacterial membrane fraction. However, it’s requirement in mycobacterial physiology was not shown until the comprehensive work done by McFadden and coworkers (Beste et al., 2009). The authors showed that the protein is uniquely required for mycobacterial growth in Roisin’s minimal media, which contains limited carbon source (Beste et al., 2009). Proteomics studies have further identified that FabG4 is one of the major proteins which expression is induced by the antibiotic stressor (Sharma et al., 2010). The protein expression was also detected in the drug-resistant strains (Singh et al., 2016; Verma et al., 2017). Most interestingly, FabG4 can functionally complement eukaryotic β-oxoacyl-ACP reductase activity. FabG4, when expressed in the knockout of *ora1* yeast cells can restore the mitochondrial fatty acid synthesis type-II (Gurvitz et al., 2009). Because yeast ORA1 is a mitochondrial FabG1/FabG4 ortholog (Hiltunen et al., 2009), functional complementation of ORA1 with FabG4 is indicative of an active FabG4 protein that can utilize ACP/Coenzyme A tagged β-oxoacyl substrates and is utilized by mycobacteria during limiting resource or antibiotic stressed condition.

## Unique Features of FabG4

**FIGURE 1:**
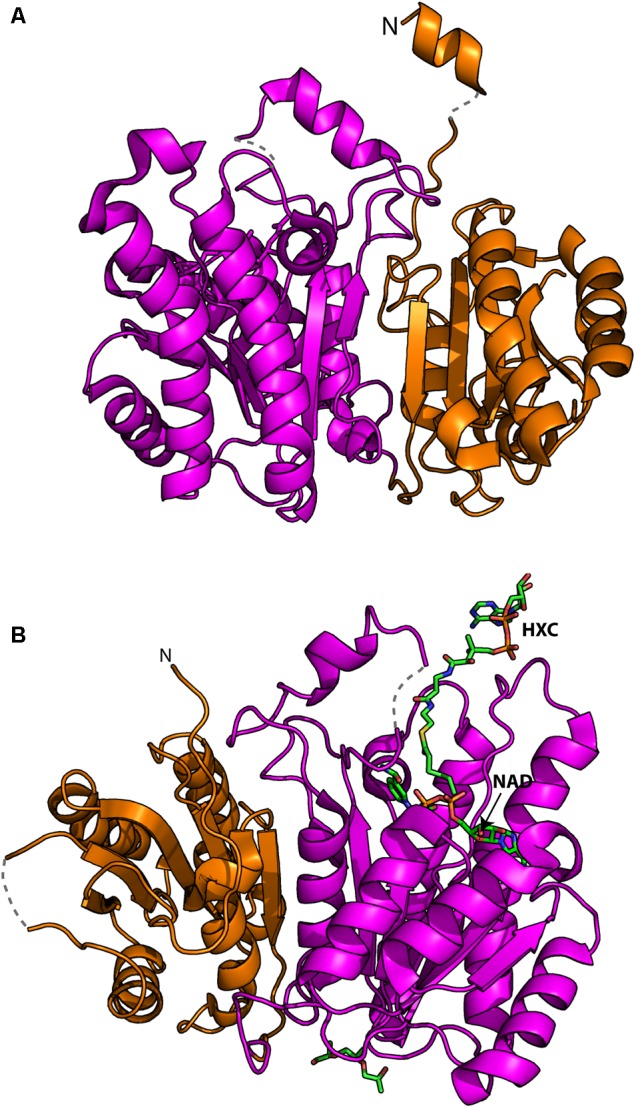
Crystal structures of FabG4: **(A)** N-terminal “flavodoxin type” (orange) and C-terminal ketoreductase (magenta) domains. “N” indicated the N-terminal of the polypeptide chain. The regions those were not be traced in electron density are shown in dotted lines. **(B)** The substrate mimic hexanoyl coenzyme A (HXC) and NAD^+^ bound structure shows the putative substrate binding modes.

Characterization of recombinantly expressed FabG4 has shown that the protein is highly specific for NADH (Dutta et al., 2011). This is intriguingly different from its close relative (33% homology) FabG1 that utilizes NADPH. Crystal structure of the FabG4 complex with NADH reveals that the specificity comes from a single aspartate residue that reduces the volume of the phosphate binding groove of adenosine ribose phosphate moiety of NADPH (Dutta et al., 2013). Crystal structure further shows that FabG4 contains a “flavodoxin-type” structural domain in its N-terminal and a typical ketoreductase domain in its C-terminal (**Figure 1A**). The N-terminal structural domain is also found in modular polyketide synthase ketoreductase domain PlmKR1 (Bonnett et al., 2013). PlmKR1 exists as a monomer but FabG4 is different in the sense that the protein is a dimer, where the N-terminal domain of a protomer makes an extensive interaction with the C-terminal domain of the other protomer. Therefore, FabG4 was designated as “High Molecular weight FabG” (HMwFabG) (Dutta et al., 2011). Typical FabG does not contain this structural domain.

Many of the N-terminal residues (1–19) of FabG4 cannot be traced in crystal structure suggesting that the region has no rigid secondary structure. Most of the N-terminal residues were traced in FabG4 complex with NAD^+^ structure (PDB 4FW8) showing a possibility of short helix containing seven residues (21–27) (**Figure 1A**). Long N-terminal sequences in FabGs are also found among eukaryotes, which were suggested to be the signal sequence (Wickramasinghe et al., 2006). Whether it is true for FabG4 is not known, but sequence alignment of HMwFabGs from actinobacteria and many proteobacteria reveals conserved residues Pro30, Leu33, and Arg35 in N-terminal sequence. Truncation of the N-terminal residues does not have any effect on catalytic activity. On the contrary, the C-terminal residues of FabG4 are apparently involved in catalytic activity since its truncation yields with a defective protein (Dutta et al., 2011). This is because the conserved C-terminal residues are engaged in electrostatic interaction with the active site proximal loops (Dutta et al., 2011).

## Inhibitors of FabG4

Structure of FabG4 and its complex with a substrate mimic hexanoyl-CoA (PDB 3V1U) accelerated the work on structure-based inhibitor design against FabG4 (Dutta et al., 2013) (**Figure 1B**). The particular structure also provides a platform to design FabG specific inhibitors because the structure provided the first evidence of FabG-substrate complex (**Table 1**). The first reported FabG4 inhibitors are based on the polyphenol compounds (Banerjee et al., 2014, 2015b). Synthesized compounds are also tested against *M*. *smegmatis* showing the MIC of 5 μg mL^-1^. Docking studies had revealed that the compounds potentially occupy the NADH binding region with a few hydrogen-bonding interactions with the loop-residues responsible for CoA substrate binding. Another study to design and synthesize the inhibitors based on the common pharmacophores like β-lactam and Isoniazid was also successful inhibiting FabG4 with IC_50_ as low as 15.2 ± 0.5 μM (Banerjee et al., 2015a). Similar to the polyphenol based compounds β-lactam and Isoniazid-based compounds primarily dock on the NADH binding site. Isothermal titration calorimetry indicates two sequential binding sites showing positive cooperativity. Inhibitory effects of β-lactam and Isoniazid-based compounds have shown to be inhibitory toward mycobacterial biofilm formation. Recently, thiophene containing trisubstituted methane compound, *S*-S006-830 was found to have a substantial inhibitory effect on *M*. *tuberculosis* biofilm formation and possesses antitubercular activity (Singh et al., 2017). FabG4 is one of the three membrane-associated proteins identified to strongly interact with *S*-S006-830. Computational docking study predicts two possible binding sites. One comprises of the region overlapping NADH and CoA substrate binding and the other site is on the N-terminal domain. *S*-S006-830 is special because apart from the NADH-substrate binding site it also targets to the N-terminal structural domain. Two unique FabG inhibitors are worth mentioning in this regard. The first, Pyridomycin analog specifically targets NADH binding proteins and proposed to exhibit inhibitory effects on FabG4 by bridging both NADH and substrate-binding region (Hartkoorn et al., 2014). The second inhibitor was identified using library screening that uniquely binds to the helical dimeric interface of FabG (Cukier et al., 2013). The helical dimeric interface in FabG is responsible of NAD(P)H binding cooperativity (Dutta et al., 2012). All FabG4 and related inhibitors are summarized in **Table 1**.

**Table 1:** Inhibitors of FabG4.

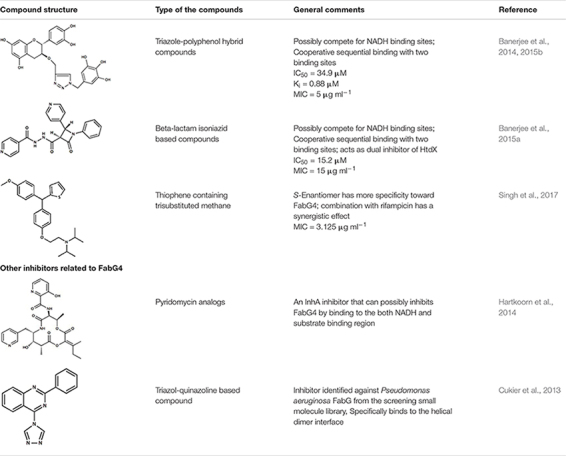

## Possible Role of the Enzyme

High Molecular weight FabG are exclusively found among bacteria. Homologous genes of FabG4 are found in actinobacteria and many proteobacteria (Dutta et al., 2011). Genomic analysis of HMwFabG containing bacteria shows another protein coexisting downstream to FabG4 ORF. In *M*. *tuberculosis* this protein is HtdX (Rv0241c). Both the proteins are conserved among mycobacterial species. Even in *M*. *leprae* genome where the genomic reduction had happened, retained both *fabG4* and its downstream *htdX* (Cole et al., 2001). However, deletion of *htdX* or *fabG4* does not show any apparent effect on bacterial survival *in vivo* (Sassetti and Rubin, 2003). Nonetheless, the protein is shown to be essential for survival in minimal media (Beste et al., 2009). Presumably, the conditional dependency of *M*. *tuberculosis* on FabG4 indicates an alternative pathway during limiting resource condition. Another clue is the FabG4 dependency on NADH. The NADH utilizing β-ketoacyl reductase activity of FabG4 is four times higher to the reverse reaction that utilizes NAD^+^ for β-hydroxyacyl dehydrogenase activity (Dutta et al., 2013). Compared to NADPH, NADH is the low energy molecule and mostly associates to the catabolic pathways (Cantó et al., 2015). Shifting the coenzyme specificity toward low energy NADH possibly indicates that the bacteria are rewiring its metabolism toward the energy saving mode. Furthermore, FabG4 is isolated from the membrane fraction, which is indicative of its membrane-associated role (Gu et al., 2003; Singh et al., 2017). The role could be either fatty acid synthesis or modification. Notably, the other gene *htdX* was found to be responsive to the inhibitors of cell wall synthesis (Boshoff et al., 2004). Since both *fabG4* and *htdX* are situated in a conserved cluster it is tempting to predict that they are involved in the same pathway. Both sequence analysis and preliminary enzymatic activities are corroborated that HtdX can commit a dehydratase reaction that is to convert β-hydroxyacyl-CoA to enoylacyl-CoA (Gurvitz, 2009; Sacco et al., 2010; Biswas et al., 2013; Banerjee et al., 2015a). The reaction is theoretically the successive step of FabG4 enzymatic reaction for fatty acid elongation cycle. Altogether the data suggests that during limiting resource condition FabG4 participates into some low-energy requiring pathway that is related to membrane biosynthesis, remodeling or recycling.

## Future Scope

FabG4 is a host antigenic protein reportedly expressed in mycobacterial biofilms. The protein expression was consistently found in membrane fraction and stressor-induced. As a conserved gene in all mycobacterial species, FabG4 can be a biomarker for biofilm. Although, FabG4 inhibitors are manifested antitubercular activity more experiments are needed to call it a drug target. Role of FabG4 and its connection with the membrane in mycobacteria also require further experimentations. To study FabG4 importance in biofilm forming *M. tuberculosis* it is, therefore, necessary to find out its targeting and interaction with other proteins. Identifying the role of FabG4 in mycobacteria might provide a hint of a new aspect of a survival strategy. The second aspect is the strategy to design more specific inhibitors of FabG4. While the C-terminal domain of FabG4 is common among FabGs, inhibitors against its N-terminal structural domain might be a way to target the protein more specifically. Designing the fluorophore inhibitors against its structural domain could be a way to find out its role *in vivo*.

## Author Contributions

DD is solely responsible for the conception or design, drafting, and revising the work.

## Conflict of Interest Statement

The author declares that the research was conducted in the absence of any commercial or financial relationships that could be construed as a potential conflict of interest.
